# Edgewise Compression and Three-Point Bending Analyses of Repaired Composite Sandwich Panels

**DOI:** 10.3390/ma16124249

**Published:** 2023-06-08

**Authors:** Ricardo J. B. Rocha, Marcelo F. S. F. de Moura, Raul D. F. Moreira

**Affiliations:** 1 INEGI—Instituto de Ciência e Inovação em Engenharia Mecânica e Engenharia Industrial, Rua Dr. Roberto Frias 400, 4200-465 Porto, Portugal; rjrocha@inegi.up.pt (R.J.B.R.); rmoreira@fe.up.pt (R.D.F.M.); 2 Departamento de Engenharia Mecânica, Faculdade de Engenharia da Universidade do Porto, Rua Dr. Roberto Frias, 4200-465 Porto, Portugal

**Keywords:** sandwich panel, scarf repair, edgewise compression, three-point bending, cohesive zone modelling

## Abstract

In this work, the fracture behaviour of repaired honeycomb/carbon–epoxy sandwich panels under edgewise compression and three-point bending loading was analysed. Assuming the occurrence of damage resulting from a complete perforation leading to an open hole, the followed repair strategy consists of plug filling the core hole and considering two scarf patches with an angle of 10° in order to repair the damaged skins. Experimental tests were performed on undamaged and repaired situations in order to address the alteration in the failure modes and assess the repair efficiency. It was observed that repair recovers a large part of the mechanical properties of the corresponding undamaged case. Additionally, a three-dimensional finite element analysis incorporating a mixed-mode I + II + III cohesive zone model was performed for the repaired cases. Cohesive elements were considered in the several critical regions prone to damage development. The failure modes and the resultant load–displacement curves obtained numerically were compared with the experimental ones. It was concluded that the numerical model is suitable for estimating the fracture behaviour of sandwich panel repairs.

## 1. Introduction

The structural application of sandwich panels is increasing due to their appealing characteristics. In fact, these components provide high stiffness and strength alongside a low weight, which is a vital aspect nowadays, regarding energy saving in moving structures, e.g., transportation and wind industries [1,2]. Nevertheless, sandwich-panel-based structures are vulnerable to damage, particularly under impact loading [3,4]. Generally, damaged large panels are rejected and replaced, which contributes to an unwanted ecological footprint and significant economic costs [5]. In these circumstances, the repair of the damaged components is required in order to extend their durability. The goal is to ensure that a large part of the mechanical properties of a damage component can be recovered after its repair, thus becoming a sustainable engineering approach [6,7].

Some researchers have dedicated their attention to the subject of sandwich repairs. Several experimental and numerical works focus on the behaviour of repaired sandwich composites with a two-dimensional configuration [8,9,10,11]. In these cases, bonded joint geometries are assumed to allow a simpler two-dimensional analysis. However, three-dimensional studies are recommended since patch repairs are usually circular, giving rise to a complex stress state under general loading. Raju et al. [12] performed experimental studies on composite sandwich panel specimens of polyurethane foam core and an aramid honeycomb core type damaged by impact loading. They carried out four-point bending and edgewise compression tests on undamaged, damaged and repaired specimens. The authors concluded that the strength recoveries revealed similar trends (values around 90%), with bending recovery showing a slight advantage compared to compression. Liu et al. [13] studied bonded repair on sandwich panels with one-side-skin and full-depth damage penetrating to the core under edgewise compression. They developed a three-dimensional progressive damage finite element model in order to predict the ultimate load, damage evolution process and stress distributions in adhesives. They studied the influence of different repair parameters including repair materials, taper ratios and repair techniques (scarf and step). The authors state that the numerical model provides an appropriate tool for the mechanical behaviour prediction of repaired sandwich panels. Ghazali et al. [14] studied the static mechanical performance of the repaired sandwich panels of a carbon–epoxy composite and honeycomb core. They performed edgewise compression and four-point bending tests on pristine and repaired panels with a stepped-scarf circular patch. A three-dimensional finite element analysis considering the different mechanical behaviours of the skins, core and adhesive was carried out. The numerical results were found to be in good agreement with the experimental ones regarding stiffness and strength predictions. Taotao Zhang et al. [15] performed experimental and numerical analyses of the damage propagation and ultimate strength of undamaged, open-hole and repaired sandwich panels under edgewise compressive load. They observed that the compressive strength of the open-hole damaged plate was about 34% of the undamaged one, increasing up to 76% for the repaired case. The numerical model accounts for intralaminar, interlaminar and honeycomb damage and was found to provide results in agreement with experimental data, thus contributing to improvements in the design and analysis techniques used to complete the scarf patch repair of sandwich structures. In this context, the model was subsequently used in parametric studies to assess the influence of the scarf angle, ply sequence and different overlaps on the ultimate strength and stress distribution in scarf-repaired sandwich plates. Yang et al. [7] studied the influence of the scarf patch repair angle on the bending strength of a honeycomb sandwich. They performed experimental and numerical analyses to evaluate the bending strength recovery. The authors concluded that the optimal solution involves using an angle of 30:1, which enables the total bending strength recovery of the sandwich structure. They also developed a three-dimensional finite element model incorporating spring elements and concluded that the model reproduces the experimental trends well.

In this work, sandwich carbon-fibre-reinforced polymer (CFRP)/honeycomb panels repaired using bonded circular patch scarf repairs were analysed under edgewise compression and three-point bending loading. Depending on the impact energy, damage can affect the outer skin, outer skin and internal core, or even cause full perforation. In this work, the most detrimental situation (i.e., full perforation) is analysed. Experimental tests on undamaged and repaired panels were performed in order to assess the influence of repair on the stiffness and strength of the panels. Three-dimensional finite element analyses involving cohesive zone modelling were employed to simulate the behaviour of the repaired panels. The results obtained revealed the suitability of the proposed methodology regarding the design predictions of the repaired sandwich panels.

## 2. Experimental Work

**Figure 1:**
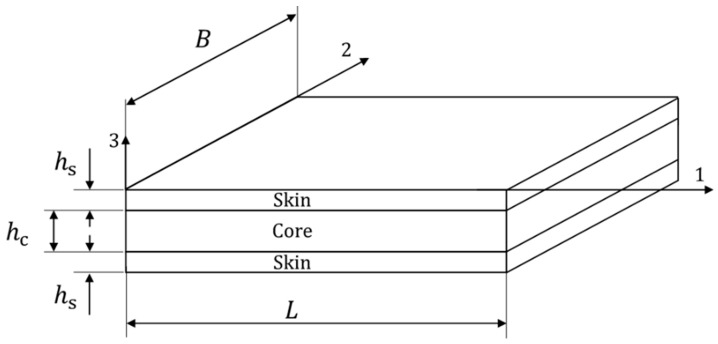
Schematic representation of the sandwich panels. Dimensions in mm: *h*_s_ = 1.35, *h*_c_ = 10; edgewise compression (*L* = 150, *B* = 100); three-point bending (*L* = 170, *B* = 250).

A sandwich panel comprising two skins of CFRP DDCFX005 (Torayca FT300-40B and DYNEEMA^®^ SK99) fabric, with a [(±45)/(±45)/(90, 0)/(±45)/(±45)] layup with a 1.35 mm thickness and a core of NOMEX^®^ Honeycomb with a 10 mm thickness, was used in this study (Figure 1). The elastic properties of the laminate were experimentally determined and are listed in Table 1. The core elastic properties are given in Table 2 [16,17].

The skins were bonded to the core using the structural epoxy adhesive ARALDITE^®^ 2015-1 from Huntsman (*E* = 1850 MPa and *ν* = 0.3). Before bonding, the surfaces of the skin were sandpapered and subsequently cleaned with isopropyl alcohol to remove the impurities and contaminants of the bonding surface, thus improving the adhesion. The undamaged specimens were obtained by bonding the skins to the honeycomb core.

**Figure 2:**
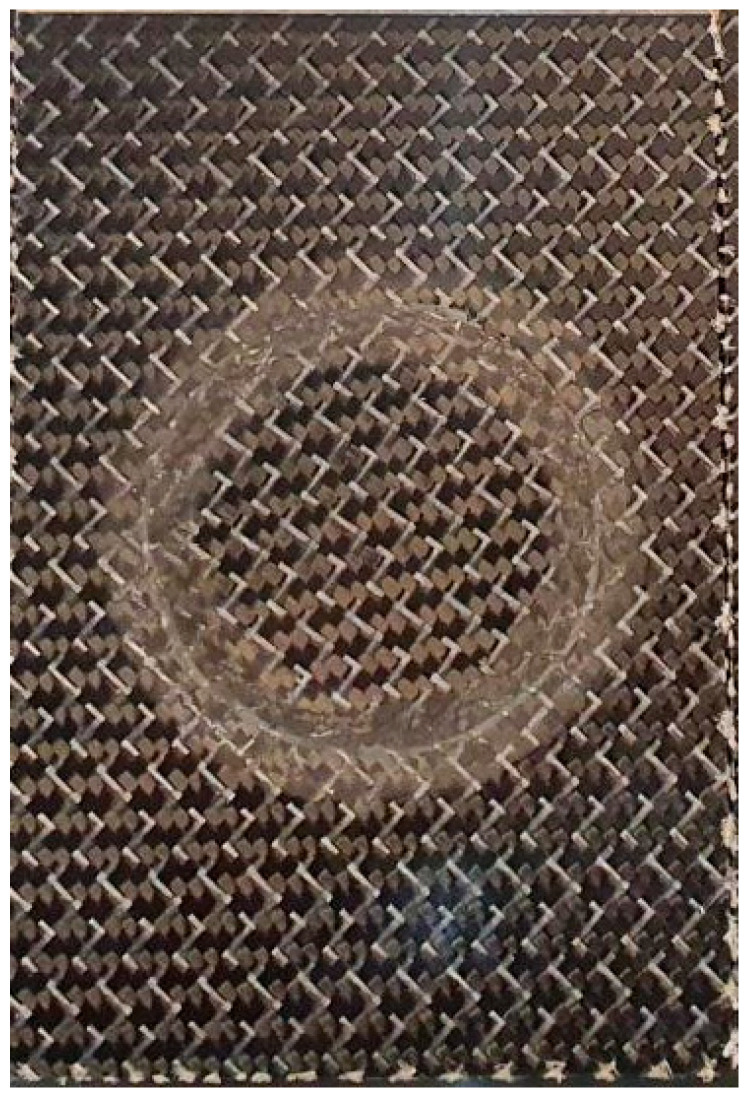
Scarf repaired specimen.

The damaged specimens were considered to suffer perforating damage induced by the high-velocity impact of a small projectile. In this context, a central hole was created, removing all the damaged region englobing the two skins and core. Additionally, two scarf chamfer profiles with an angle of 10° were machined on both skins using a CNC milling machine. Afterwards, a repair scheme was adopted with the aim of restoring a substantial part of the initial stiffness and strength. A cylindrical piece of core was bonded with ARALDITE^®^ 2015-1 inside the core hole to replace the damaged and removed part of the core. Finally, two scarf patches manufactured with the same angle of 10° were bonded to both skins and to the core plug. It should be noted that both the parent laminate and patch were sandpapered (120-grit) in the scarf region to promote good adhesion. A constant adhesive thickness of 0.2 mm was assured using a calibrated wire located between the patches and the skins during the bonding process. The main characteristic of scarf repairs is the fact that patches are entirely inserted in the panel, thus not altering its aerodynamic behaviour (Figure 2).

**Figure 3:**
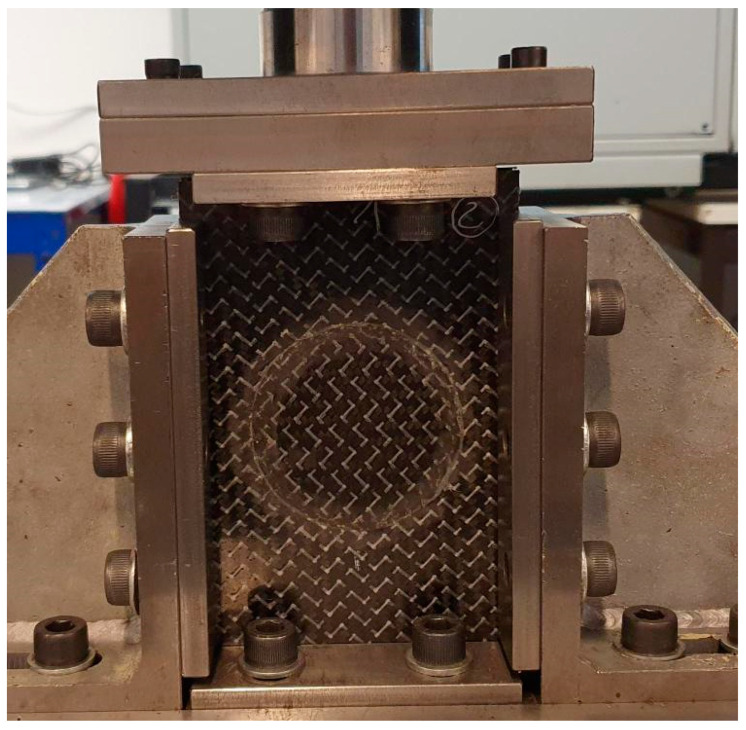
The experimental setup of the edgewise compression tests.

**Figure 4:**
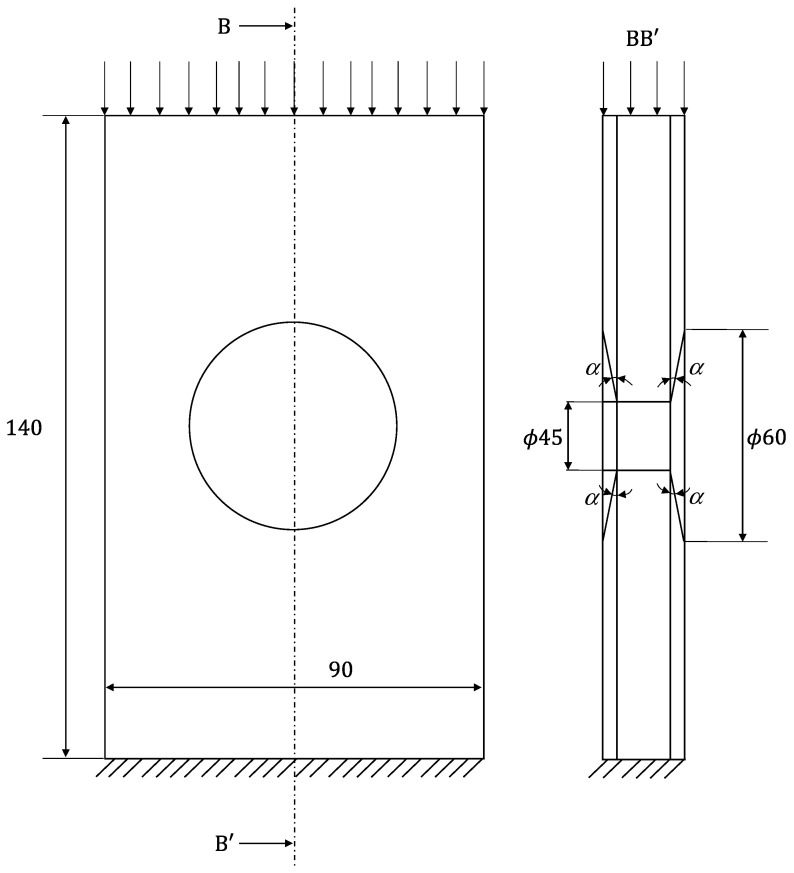
Schematic representation of the edgewise compression test (*α* = 10°).

Two different experiments were performed in this work: edgewise compression and three-point bending tests. For the edgewise compression tests, the procedure described in the ASTM D7137 standard was followed [18] and a special device with anti-buckling guides was used (Figure 3). The useful specimen dimensions are shown in Figure 4.

**Figure 5:**
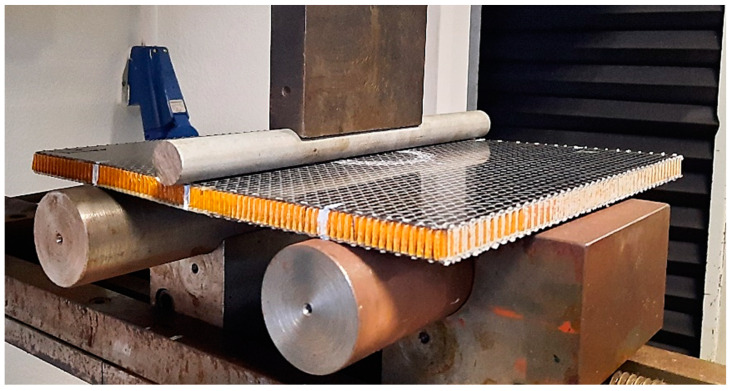
The experimental setup of the three-point bending tests.

**Figure 6:**
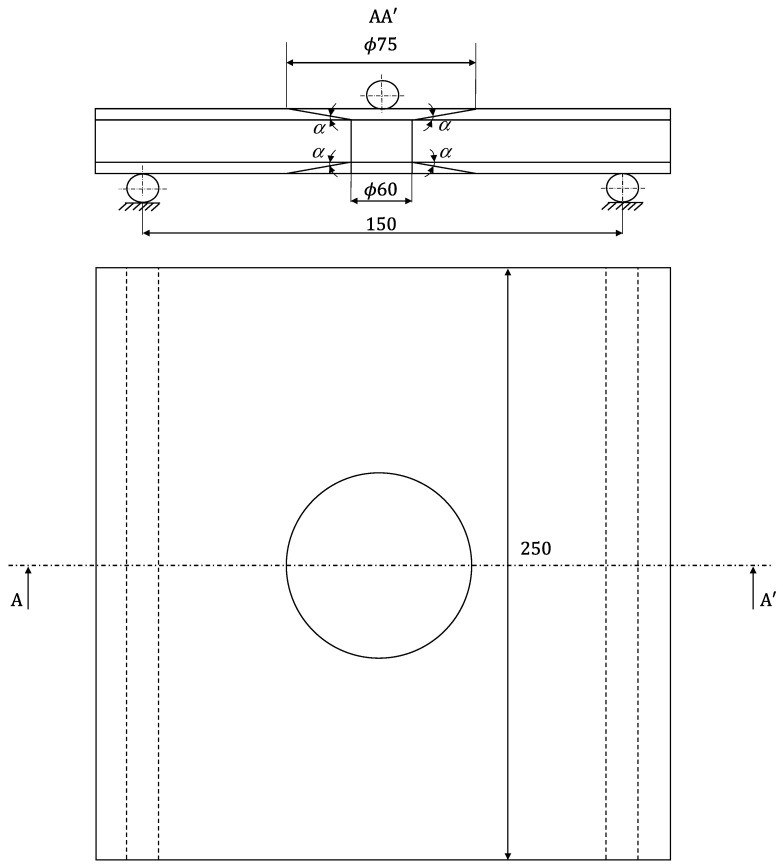
Schematic representation of the three-point bending test (*α* = 10°).

For the three-point bending tests (Figure 5), an adaptation of the ASTM D790 standard was used [19]. Owing to the specimens’ dimensions, a bigger damage region was considered (Figure 6) when compared to the compression tests, since previous studies have revealed that bending is less influenced by damage when compared to compressive behaviour.

In both cases, the tests were performed under displacement control with a rate of 1 mm/min using a universal testing machine (INSTRON^®^ 5900R) equipped with a load cell of 20 kN. The load–displacement (*P*-*δ*) curves were registered for subsequent analyses focusing on the evaluation of the initial stiffness and strength of the tested panels.

## 3. Numerical Analysis including CZM

**Figure 7:**
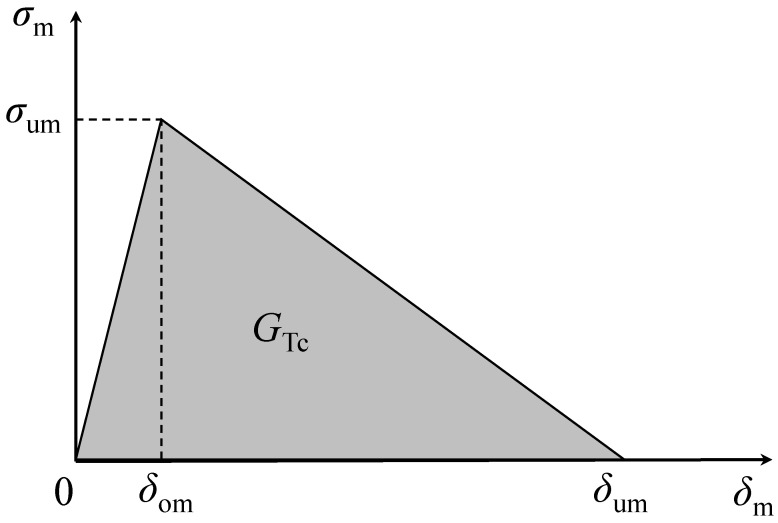
The linear softening cohesive law under mixed-mode I + II + III loading (subscript m): *δ*_om_—damage onset relative displacement; *δ*_um_—ultimate relative displacement; *σ*_um_—local strength; *G*_Tc_—fracture energy.

Three-dimensional finite element analyses, including cohesive zone modelling, were performed considering the edgewise compression and three-point bending tests applied to the repaired panels. Owing to the development of complex loading at the repair region, a mixed-mode I + II + III damage model [20], considering the linear softening law, was used (Figure 7). The quadratic stress criterion is considered to deal with damage onset:

(1) $${\left(\frac{\sigma_{I}}{\sigma_{u,I}}\right)}^{2}+{\left(\frac{\sigma_{\mathrm{II}}}{\sigma_{u,\mathrm{II}}}\right)}^{2}+{\left(\frac{\sigma_{\mathrm{III}}}{\sigma_{u,\mathrm{III}}}\right)}^{2}=1$$

where ($\sigma_{I},\;\;\sigma_{\mathrm{II}},\;\;\sigma_{\mathrm{III}}$) are the mode I, II and III traction components, respectively, and ($\sigma_{u,I},\;\;\sigma_{u,\mathrm{II}},\;\;\sigma_{u,\mathrm{III}}$) are the corresponding local strengths. Damage propagation was simulated considering the linear energetic criterion:

(2) $$\left(\frac{G_{I}}{G_{\mathrm{Ic}}}\right)+\left(\frac{G_{\mathrm{II}}}{G_{\mathrm{IIc}}}\right)+\left(\frac{G_{\mathrm{III}}}{G_{\mathrm{IIIc}}}\right)=1$$

where *G_i_* and *G_i_*_c_ (*i* = I, II, III) represent the strain energy release rate components and the corresponding critical values, respectively. After damage initiation (Equation (1)), a damage parameter (*d*) must be considered in order to mimic material softening:

(3) $$\sigma_{m}=\left(1-d\right)k\delta_{m}$$

where *k* is the interfacial stiffness, $\delta_{m}$ is the equivalent mixed-mode I + II + III relative displacement ($\delta_{m}=\sqrt{\delta_{I}^{2}+\delta_{\mathrm{II}}^{2}+\delta_{\mathrm{III}}^{2}}$), and $\sigma_{m}$ is the corresponding mixed-mode I + II + III traction. After some algebraic manipulations [20], Equations (1) and (2) can be used to obtain the equivalent mixed-mode displacements at damage onset ($\delta_{\mathrm{om}}$) and at failure ($\delta_{\mathrm{um}}$), according to Figure 7. These parameters are used to define the damage parameter

(4) $$d=\frac{\delta_{\mathrm{um}}\left(\delta_{m}-\delta_{\mathrm{om}}\right)}{\delta_{m}\left(\delta_{\mathrm{um}}-\delta_{\mathrm{om}}\right)}$$

that is used in Equation (3) to simulate material stiffness reduction.

**Figure 8:**
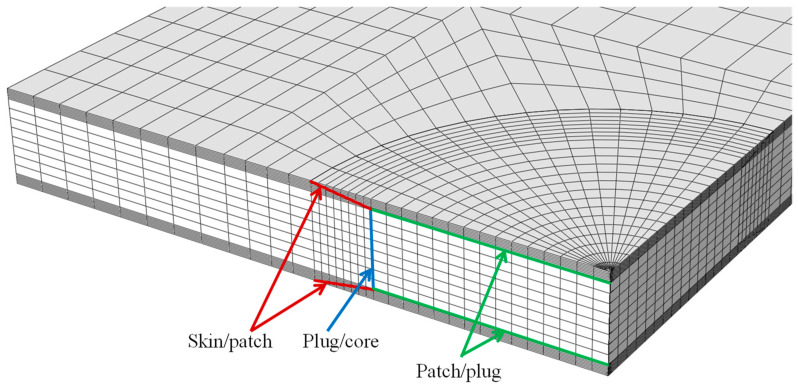
Location of cohesive zone elements.

The cohesive zone (CZ) elements were located at the critical regions prone to damage development: skin/patch in the scarf region, patch/plug, and plug/core interface planes (Figure 8).

## 4. Results

### 4.1. Edgewise Compression Tests

**Figure 9:**
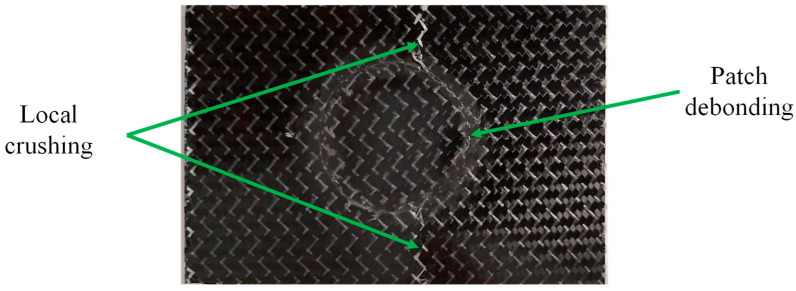
Typical damage obtained in edgewise compression tests of repaired specimens.

Experimental edgewise compression tests, considering an undamaged plate and four repaired ones, were performed. The undamaged case fails due to local crushing at its extremities (loaded and supported). The failure mode of the repaired specimens involves patch debonding followed by local crushing at the specimen mid-plane, which is caused by a reduction in the resistant section (Figure 9). The patch debonding reflects on a peak load that was assumed to be representative of the specimen strength.

**Figure 10:**
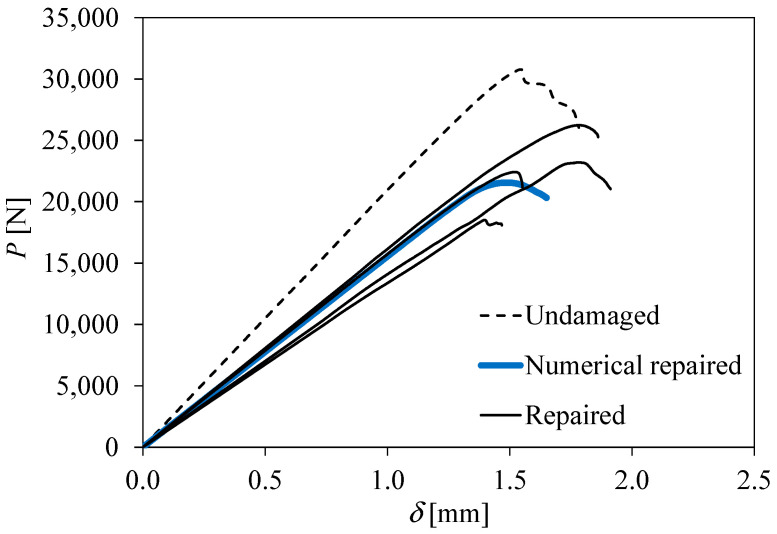
Load–displacement curves of the edgewise compression tests.

The load–displacement curves of the edgewise compression tests are presented in Figure 10. It can be observed that the initial stiffness and strength were not completely recovered after repair. Considering all the results, the initial stiffness and strength are in the range of 70–75% of the undamaged case. If we discard the lowest stiffness and strength case, assuming that it is an outlier result induced by any imperfection, the previous results change to the range of 75–80% of the undamaged case.

The three-dimensional numerical model considering the finite element analysis includes 22,016 solid elements (eight-node brick and six-node wedge) and compatible eight-node cohesive elements with null thickness. Only a quarter of the plate was simulated owing to symmetry conditions. A displacement (*δ*) was applied to the plate upper boundary to induce compressive loading, using small increments (0.02 mm per increment) in order to ensure stable damage development. The cohesive parameters used in the simulations were determined in previous works [16,17,21] and are listed in Table 3 and Table 4.

**Figure 11:**
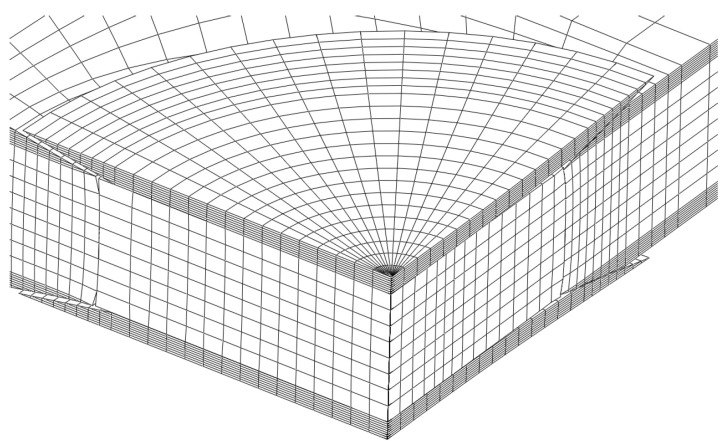
Damage obtained numerically in the edgewise compression tests.

Figure 11 reveals the damage profile obtained numerically. As observed experimentally, patch debonding occurs, which defines the specimen strength. The numerical load–displacement curve is included in Figure 10, demonstrating that it represents the overall experimental trend well.

### 4.2. Three-Point Bending Tests

**Figure 12:**
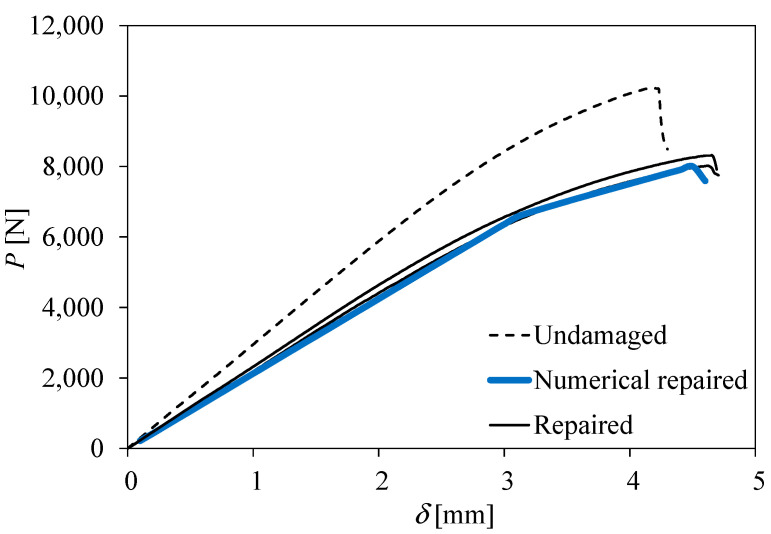
Load–displacement curves of the three-point bending tests.

**Figure 13:**
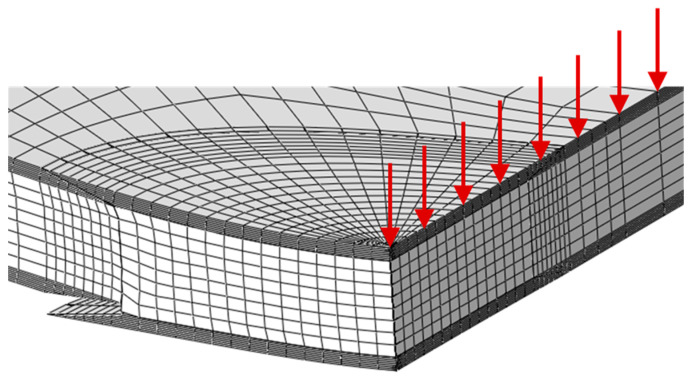
Damage obtained numerically in the three-point bending tests.

Owing to premature failures, only two three-point bending measurements of the repaired specimens were obtained. In addition, both load–displacement curves are quite consistent with each other (Figure 12), which validates their consideration. In both cases, patch debonding of the external patch relative to the loaded surface (Figure 13) leads to a peak load that defines the specimen strength. Some non-linearity can be observed before the peak load, which can be explained by the development of non-negligible fracture process zones under mixed-mode loading. Similar to what happened in the edgewise compression tests, the stiffness and strength of the repaired plates are in the range of 75–80% of the undamaged case. In this type of test, the failure mode was dictated by the localised crushing of the upper skin at the specimen mid-span due to compressive stresses induced by bending.

The numerical analysis was performed considering the same mesh and material properties used in the edgewise compression tests. The unique alterations consider the dimensions of the specimen and of the patch repair. A loading displacement (red arrows in Figure 13) was applied in the symmetry plane (specimen mid-span) by using small increments (0.02 mm) in order to avoid numerical instabilities. As observed experimentally, the failure mode occurs by the debonding of the external patch relative to the loaded surface, which allows the specimen strength to be defined. The numerical load–displacement curve reflects the experimental results well, revealing that the proposed model is adequate in order to simulate different repair strategies in three-dimensional progressive damage analysis.

## 5. Conclusions

In this work, the efficiency of scarf repairs on honeycomb/carbon–epoxy sandwich panels damaged by complete perforation (open-hole damage) was addressed experimentally and numerically under edgewise compression and three-point bending loading. The repair scheme is based on the plug filling of the damaged core with honeycomb and the use of two scarf patches with an angle of 10° in order to repair the damaged skins. In both loading cases, it was verified that the initial stiffness and strength of the corresponding undamaged situation were recovered up to 75–80% after repair. The main reason for this is the alteration of the failure mode which, in the repaired case, is mainly dictated by patch debonding that subsequently triggers the final collapse of the panel.

A three-dimensional numerical model based on finite element analysis, including mixed-mode I + II + III cohesive zone modelling, was developed for the repaired cases. Cohesive elements were considered in the critical regions prone to damage onset. The experimental failure modes were captured well by the model. The numerical load–displacement curves reproduce the observed experimental trends in both cases well. It was concluded that the model could be considered a useful tool regarding the optimization of the design of repairs for sandwich panels.

## Figures and Tables

**Table 1:** Elastic properties of CFRP.

*E*_1_ = 14,400 MPa	*ν*_12_ = 0.0017	*G*_12_ = 3550 MPa
*E*_2_ = 14,400 MPa	*ν*_13_ = 0.04	*G*_13_ = 2070 MPa
*E*_3_ = 2000 MPa	*ν*_23_ = 0.04	*G*_23_ = 2070 MPa

**Table 2:** Elastic properties of the NOMEX^®^ Honeycomb [16,17].

*E*_1_ = 0.45 MPa	*ν*_12_ = 0.9956	*G*_12_ = 0.11 MPa
*E*_2_ = 0.45 MPa	*ν*_13_ = 0.0005	*G*_13_ = 38.62 MPa
*E*_3_ = 258 MPa	*ν*_23_ = 0.0005	*G*_23_ = 63.12 MPa

**Table 3:** Cohesive parameters used for skin/core debonding analysis [16,17].

$\sigma_{u,I}$ (MPa)	$\sigma_{u,\mathrm{II}}$ (MPa)	*G*_Ic_ (N/mm)	*G*_IIc_ (N/mm)
1.0	1.5	0.39	1.0

**Table 4:** Cohesive parameters used for scarf debonding analysis [22].

$\sigma_{u,I}$ (MPa)	$\sigma_{u,\mathrm{II}}$ (MPa)	*G*_Ic_ (N/mm)	*G*_IIc_ (N/mm)
18.0	25.0	0.49	4.59

## Data Availability

The data presented in this study are available on request from the corresponding author. The data are not publicly available due to privacy.
